# Randomized controlled trial on the treatment of Hypoferritinemia without Anemia: Comparing oral and intravenous iron supplementation among reproductive age women in Pakistan

**DOI:** 10.12669/pjms.41.1.11083

**Published:** 2025-01

**Authors:** Shehla Akram, Abdul Majeed Akhtar, Rubeena Zakar

**Affiliations:** 1 Dr. Shehla Javed Akram, MBBS, DTM & H, MPhil Public Health, University Institute of Public Health, Faculty of Allied Health Sciences, The University of Lahore, Lahore, Pakistan; 2 Dr. Abdul Majeed Akhtar, MBBS, PhD Public Health, University Institute of Public Health, Faculty of Allied Health Sciences, The University of Lahore, Lahore, Pakistan; 3 Dr. Rubeena Zakar, MBBS, PhD Public Health, Department of Public Health, Institute of Social and Cultural Studies, University of Punjab, Lahore, Pakistan

**Keywords:** Hpoferritinemia without anemia, Serum ferritin, Ferric carboxymaltose, intravenous iron, Iron III Hydroxide Polymaltose Complex, females, Randomized, Multi-arm, Intervention, Treatment

## Abstract

**Background & Objectives:**

Hypoferritinemia without anemia (HWA) is an under-recognized public health concern. Early identification and targeted treatment of HWA can prevent unnecessary medication use and potential drug abuse. This study aims to establish clearer guidelines for recognizing and managing HWA, improving patient’s outcome. The study objectives will be to compare the treatment efficacy of oral and IV iron on patients with HWA and to check the severity of clinical indicators of HWA post treatment among reproductive age women.

**Methods:**

We will choose a multicenter, multi-arm randomized controlled trial with a parallel assignment interventional model. About three hundred participants (n=300) aged 18-45 with hypoferritinemia without anemia and no comorbidities will be recruited. Participants will be randomized into three groups of one hundred each: Group-A will receive oral iron supplements (Iron III Hydroxide Polymaltose Complex, 100 mg elemental iron) one tablet per day for three months; Group-B will receive intravenous iron (Ferric carboxymaltose, one dose per month for three months); Group-C will receive no treatment. All groups will be followed for four months.

**Conclusion:**

The primary outcome will be the change in serum ferritin levels among groups at four months post-intervention. Secondary outcomes will include changes in clinical symptoms post-intervention. Data will be analyzed by using independent t-tests for between-group ferritin levels, paired t-tests for within-group comparisons, Wilcoxon Rank and Friedman tests for clinical symptoms, and ANOVA for comparisons across multiple groups.

## INTRODUCTION

Iron Deficiency Anemia (IDA) is a widely recognized condition affecting over one billion people globally, however Hypoferritinemia without Anemia (HWA) is estimated to be at least twice as prevalent.^1^ In clinical practice, hypoferritinemia without anemia can be ascertained in adults (ages 15 to 45) when hemoglobin levels are normal (≥12 g/dl) but ferritin levels dip below 30 ng/ml.^1^ According to a study conducted on iron treatment among healthy males and females aged >15 years, a ferritin cut-off of 30 ng/ml is recommended.^2^,^3^ Another study on iron deficiency without anemia shows that the serum ferritin concentration (cut off <30 ng/ml) is the most specific test used for the identification of iron deficiency.^4^ The common symptoms of HWA include fatigue, poor work productivity, cognitive difficulties, hair loss, palpitations, sore tongue, restless leg syndrome, and poor skin, hair, and nail health.^5^,^6^ Despite its prevalence, HWA is frequently overlooked by clinicians, likely due to suboptimal screening practices and reliance on hemoglobin levels alone for diagnosis.^7^ Some patients with HWA may have had profound symptoms for many years while their blood count has been normal throughout.^8^ The normal Complete Blood Count (CBC) results make HWA a hidden disease with a lack in exact underlying cause.^9^ Similar symptoms are experienced by patients suffering from iron deficiency anemia (IDA) and HWA however, the sufferers of the latter condition suffer from wrong or no diagnosis as compared to IDA which is easily diagnosed through complete blood count (CBC).^9^ Because the symptoms and signs of HWA are not specific, which may occur in other physiological or psychiatric conditions, patients with HWA are either suggested no tests or can result in expensive tests for heart or renal failure as a result of complication of chronic iron deficiency,^5^,^10^,^11^ which may be avoidable if simple, cost-effective tests such as serum ferritin, folic acid, and vitamin B12 are conducted on time.^12^,^13^ HWA presents a diagnostic challenge as it mimics the symptoms of IDA, yet remains undiagnosed due to normal hemoglobin and CBC results.^5^ Clinicians generally recommend iron assessments when there has been evidence of anemia, resulting in undetected HWA.^4^ The clinician typically does not make an effort to identify the underlying cause of the symptoms, with the exception of chronic disease study, and instead prescribes symptomatic treatment, which might lead to drug misuse or overmedication e.g., pain killers, anti- depressants or anti-anxiety drugs, sleeping tablets and overdose of multivitamins.^14^-^15^ This study aims to bring attention to the under-recognition of HWA, advocating for better diagnostic protocols and targeted treatments to the clinicians. This study has to be carried out because not only it will help patients determine the category of HWA, but it will also assist them to obtain specific treatment, reducing their risk of drug misuse and overmedication. To date, no study on HWA has been conducted in Pakistan on non-pregnant women, highlighting the need for research in this population.

### Research Objectives:

1. To compare the effects of` oral iron (Iron III Hydroxide Polymaltose Complex eq. to Elemental Iron, 100 mg per day for three months) supplementation (Group-A) versus Intravenous (IV) iron (Ferric carboxymaltose 500 mg, three doses for three months) (Group-B) versus no treatment (Group-C) on serum ferritin level, four months post-intervention.
2. To evaluate the change in the severity of clinical indicators of Hypoferritinemia without Anemia between Group A, B and C, four months post-intervention.

## METHODS

### Study Design:

This single-blinded, multicenter, multi-arm, randomized controlled trial (RCT) will employ a parallel assignment interventional model.

### Setting:

Participants will be recruited from four major tertiary care hospitals and surrounding communities in Lahore. A comprehensive list of government and private hospitals will be obtained from the Health Department of Punjab, sorted by bed capacity. The top two private and two government hospitals will be selected based on their number of beds.

### Sample Size:

The sample size will be calculated under the hypothesis that intravenous (IV) treatment will more effectively alleviate symptom scores compared to oral or no intervention after four months. Based on a 41.7% prevalence of HWA^16^ and an anticipated 45% reduction in prevalence post-intervention, a sample size of ninety-six patients per group is required, with a 5% inflation for loss to follow-up. Therefore, one hundred patients will be recruited in each of the three groups, totaling three hundred participants.

### Eligibility Criteria:

Eligible participants will be healthy females of reproductive age diagnosed with hypoferritinemia without anemia, with no comorbidities.

**Table-I:** Study Schedule.

Schedule of clinical assessments and procedures in the hospital						

In-hospital assessment	Visit 0	Visit 01	Visit 02	Visit 03	Visit 04	Visit 05
Informed consent	Yes					
Eligibility criteria	Yes	Yes				
Baseline data	Yes					
Blood sample	Yes	Yes				Yes
Randomize			Yes			
Give study medication			Yes	Yes	Yes	
Adverse event monitoring			Yes	Yes	Yes	
Post-intervention data						Yes

### Inclusion Criteria:

- Females of reproductive age 18-45 years old
- Having iron deficiency symptoms such as fatigue/ laziness, poor productivity at work places, attention deficit and forgetfulness, sore tongue, dullness of skin and damage of nails, hair loss, prolonged wound healing and restlessness in legs
- Hemoglobin higher than or equal to 12g/dl
- Ferritin less than 30ng/ml in serum
- Females with normal range of thyroid and blood sugar

### Exclusion Criteria:

- Premature menopause
- Anemia with hemoglobin <12 g/dl or abnormal CBC, Serum ferritin level >30, disturbed thyroid or blood sugar levels
- History of iron or multivitamin consumption in the previous quarter
- Pregnant or lactating females
- Blood transfusion or donation within the last three months
- Comorbidities such as gluten intolerance, peri-anal vascular swelling, malignancies, urinary bleeding, cardiac and kidney failure, chronic gastritis, cirrhosis, or diabetes
- Concomitant Vitamin B12 or folic acid deficiencies, or inflammatory conditions affecting serum ferritin.

### Laboratory Tests:

***Complete Blood Count (CBC):*** All samples will undergo CBC to evaluate hematological parameters, including hemoglobin, red cell count, white cell count, platelet count, packed cell volume, mean cell volume, mean cell hemoglobin, mean cell hemoglobin concentration, and red cell distribution width, using an automated analyzer (Sysmex XN-1000, Tokyo, Japan).

***Serum Ferritin Concentration:*** A solid-phase sandwich assay using the streptavidin-biotin principle will be employed. Briefly, 96 wells will be covered with 200 µL of mouse anti-ferritin antibody. Serum samples, controls, and calibrators will be added in duplicate, followed by a conjugate working solution. After incubation and washing, TMB substrate will be added, and the optical density will be assessed at 450 nm with a microplate reader.

### Recruitment Strategy

### Participants will be recruited in three steps:

***Step-1:*** Purposive sampling of individuals who provide informed consent and meet the serum ferritin and hemoglobin criteria.

***Step-2:*** Screening based on inclusion and exclusion criteria, including blood tests for thyroid function and blood sugar.

***Step-3*:** Random assignment to groups A, B, or C via simple random sampling using a computer-generated list.

### Intervention:

***Group-A (n=100):*** Participants will receive oral iron supplementation (Iron III Hydroxide Polymaltose Complex, 100 mg) daily for three months, with compliance monitored through follow-up calls. Blood samples will be collected at the end of treatment period of four months.

***Group-B (n=100):*** Participants will receive IV iron supplementation (Ferric Carboxymaltose, 500 mg) once a month for three months. Blood samples will be collected at the end of treatment period of four months.

***Group-C (n=100):*** Participants will receive no treatment for three months. Blood samples will be collected at the end of treatment period of four months.

Randomized Controlled Trial Steps

### The trial will follow seven steps guided by the CONSORT guidelines:

***Recruitment of participants (Visit 0, Week 1):*** Study participants will be selected after signing the informed consent and meeting the eligibility criteria of the study. Blood sample for CBC and serum ferritin will be taken by the phlebotomist.

***Baseline data (Visit 0, Week 1):*** Baseline data will be recorded for eligible participants. The baseline data will include the socio-demographics information of the study participants and history of clinical symptoms of iron deficiency (pre intervention).

***Assessment of Eligibility (Visit 1, Week 1):*** Participants will be screened based on the results of CBC and serum ferritin. Blood samples of eligible participants will be collected for blood sugar and thyroid functions.

***Randomization (Visit 2, Week 2):*** Eligible participants with normal range of thyroid and blood sugar levels will be randomized to any of the three groups by using simple random sampling.

***Allocation (Visit 2, Week 2):*** After randomization, participants will be assigned to Groups A, B, or C, with specific intervention outlined.

### Intervention:

***Visit 2 (Week 2):*** First dose of oral or IV iron will be administered, and adverse effects will be monitored.

***Visit 3 (Week 6):*** Second doses will be administered; compliance and adverse effects will be monitored.

***Visit 4 (Week 10):*** Third doses will be administered; compliance and adverse effects will again be monitored.

***Post-Intervention Data Collection (Visit 5, Week 16):*** The participants from all three study groups will be contacted. Blood samples have to be collected to measure the efficacy of iron treatment on ferritin levels. The symptoms severity levels will also be recorded again to assess the effectiveness of the intervention.

***Compliance:*** To enhance compliance, participants in Group-B will receive daily reminder messages or calls regarding their supplementation regimen.

**Fig-1:**
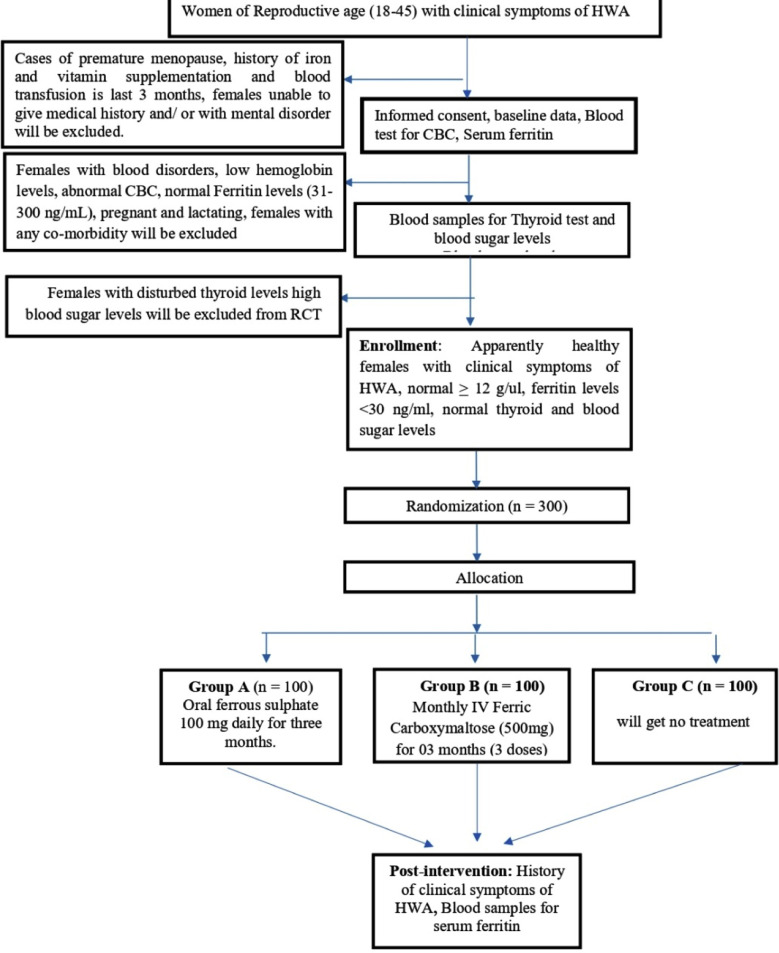
Framework of Randomized controlled trial Framework for randomized controlled trial is illustrated in Fig. 1 (also attached separately).

***Informed consent in English***

## CONSENT FORMS

**Description of the Research and Your Participation**

**You are invited to participate in a research study conducted by Dr. Shehla Akram**

**The purpose of this research is to evaluate the “**effect of oral and intravenous iron on the severity of symptoms and serum ferritin levels in patients with Hypoferritinemia without Anemia.**”**

**Risks and Discomforts**

Fever and discomfort at the site of injection/phlebotomy

**Potential Benefits**

This study will help to establish effect of oral and IV iron in the reduction of symptoms of Hypoferritinemia without anemia.

**Protection of Confidentiality**

We will do everything we can to protect your privacy. Your identity will not be revealed in any publication resulting from this study.

**Voluntary Participation**

Your participation in this research study is voluntary. You may choose not to participate and you may withdraw your consent to participate any time. You will not be penalized in any way should you decide not you participate or to withdraw from this study.

## CONSENT

**I have read this consent form and have been given the opportunity to ask questions. I give my consent to participate in this study.**

Participant’s Signature __________________ Date: ____________________

A copy of this consent form should be given to the participant.


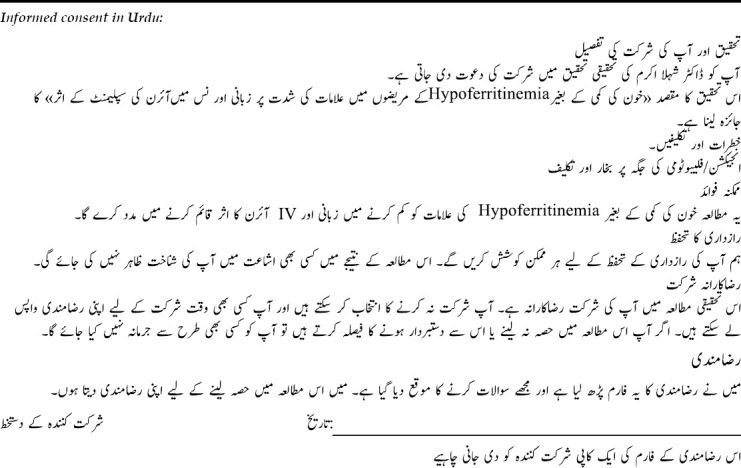


**Data Collection Tool**

Respected participant, the objective behind this study is to create awareness among clinicians to investigate the patients with above symptoms and recognize this category of HWA and treat it with iron therapy instead of symptomatic treatment and misuse of the drugs. Participation in this Survey is voluntary and you can choose not to answer any individual question. The information you provide will be kept strictly confidential and will only be used for research purposes.

Participant’s Name: _____________________________________________________

Participant’s Code: _____________________________________________________


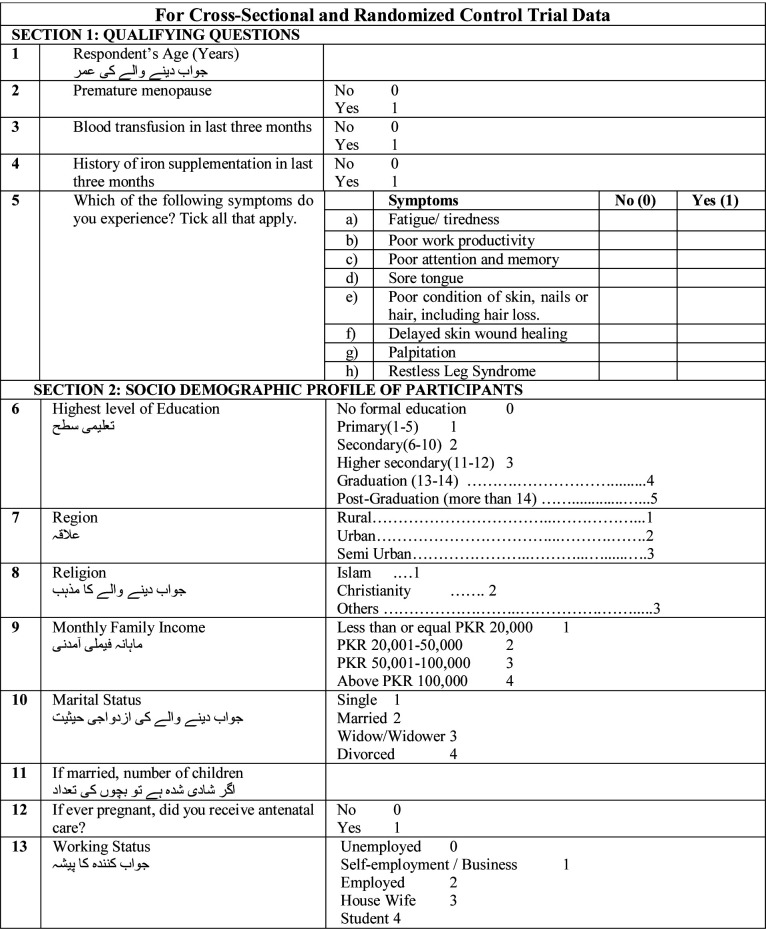



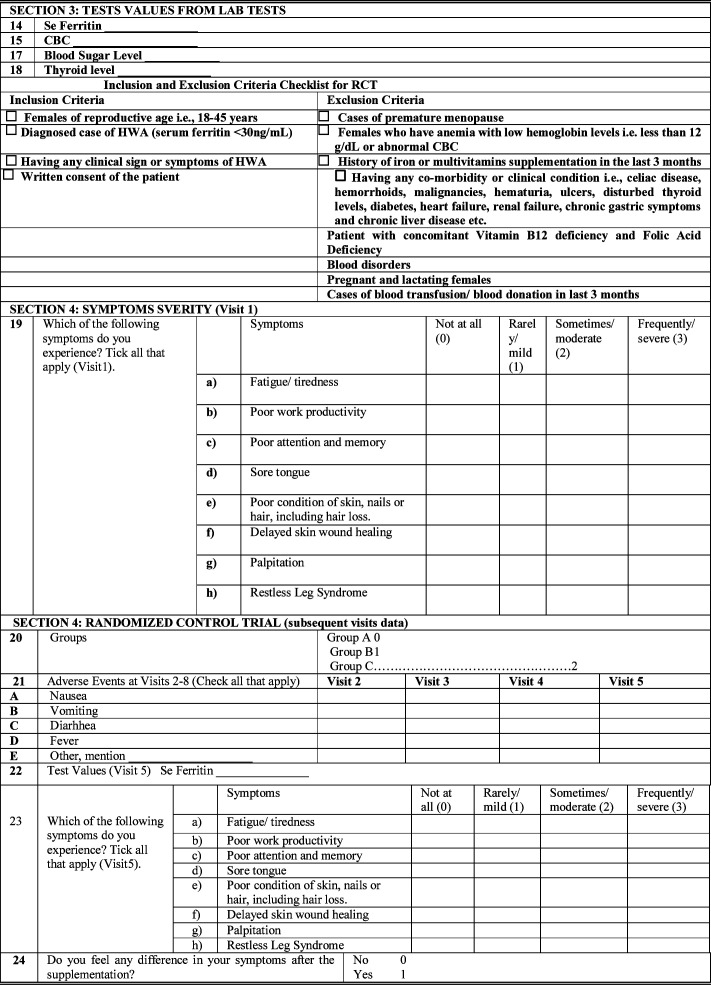


### Outcome Measures:

### Primary Outcome:

The primary outcome will be change in serum ferritin levels from baseline to Visit five. This will be measured by analyzing the difference in the treatment outcome of oral iron supplementation Group-A, intravenous iron supplementation Group-B and no treatment Group-C, four months post-intervention. A 5ml blood sample for measuring serum ferritin levels will be drawn by the phlebotomist at visit five to access the change in the value of serum ferritin from baseline.

### Secondary Outcome:

The secondary outcome will be change in severity of clinical symptoms of hypoferritinemia without anemia. The secondary outcome will be measured by analyzing the difference in the severity of clinical symptoms of hypoferritinemia without anemia in oral iron supplementation Group-A, intravenous iron supplement Group-B and no treatment Group-C, four months post-intervention. A detailed history of clinical symptoms of hypoferritinemia without anemia will be taken by the research assistant in order to assess the severity of clinical symptoms post intervention.

### Statistical Analysis:

SPSS version 24 will be used for data analysis. Descriptive and inferential statistics will be used. Comparisons will be made using independent sample t-tests, paired sample t-tests, Wilcoxon rank tests, Friedman tests, and ANOVA, as appropriate based on normality assessed by the Shapiro-Wilk test.

### Reporting of Adverse Events:

All adverse events will be documented. Patients will be monitored for immediate (within 30 minutes) and late adverse events via a 24-hour helpline at Akram Medical Complex, +42 35 710406. The helpline number leads directly to reception, where professional staff will take the call. If a patient reports an adverse event, the receptionist will immediately notify Dr. Faisal Zafar, the study doctor, who will assess the situation and provide any necessary medical advice or action. The adverse events that could reasonably arise will not be reported as primary or secondary outcomes.

### Ethical Considerations:

This randomized controlled trial is the part of the dissertation project of the first author on the topic of “Hypoferritinemia without anemia among reproductive age females: Prevalence, determinants and treatment outcomes” and is checked and approved by the Research Ethics Committee (ERC) of the University of Lahore under the reference number (REC-UOL-248-10-2022). The study has been approved by the National Bioethics Committee (NBC-887) and relevant IRBs. This study adheres to the ICH-GCP guidelines, and the trial is registered with ClinicalTrials.gov (NCT06437080). Informed consent will be taken, and confidentiality will be maintained. The personal information will be kept private and will not be shared without permission and anonymity will be assured. The participants will be assured that there will be no potential harms associated with the study procedures. Participants will be free to withdraw at any time, and their rights will be respected throughout the study.

The final results of the trial will be communicated with participants, medical experts, and different research institutions through a publicly available database and published in a peer-reviewed journal after completion. Following the trial, results will also be made available on clinicaltrials.gov. Data confidentiality will be strictly maintained; only the principal investigator will have accessibility to the data, ensuring that research assistants, investigators, and physicians cannot access participant information.

## DISCUSSION

The literature reveals a significant gap in understanding various aspects of hypoferritinemia without anemia (HWA). Firstly, HWA is often underdiagnosed due to its nonspecific symptoms, which can overlap with other conditions. While there are estimates regarding the disease burden, precise figures are lacking, particularly in developing countries. Secondly, while extensive data exist on the causes of iron deficiency, there is a paucity of information on the determinants and causes of HWA specifically. Thirdly, management strategies for HWA are contentious; some researchers advocate for oral iron as the initial treatment, whereas others support intravenous (IV) therapy to alleviate symptoms more rapidly. This trial aims to evaluate the effectiveness of oral versus IV iron by comparing ferritin levels before and after treatment. In summary, there is limited evidence-based treatment available for the clinical symptoms associated with HWA, with many patients being prescribed antidepressants or analgesics instead of appropriate iron therapy. This study’s primary objective will be to raise awareness among clinicians about the importance of diagnosing HWA in patients presenting with relevant symptoms and to encourage the appropriate use of iron therapy (oral or intravenous) rather than relying on symptomatic treatments or misuse of drugs.

### Authors’ Contribution:

**SJK:** Conceived, designed, collected data, and did statistical analysis, writing and editing of manuscript, is responsible for integrity of research.

**AMA & RZ:** Critical review, literature search, final approval of manuscript.
